# Melatonin Suppresses Oral Squamous Cell Carcinomas Migration and Invasion through Blocking FGF19/FGFR 4 Signaling Pathway

**DOI:** 10.3390/ijms22189907

**Published:** 2021-09-14

**Authors:** Leilei Wang, Yuxiong Su, Wing Shan Choi

**Affiliations:** Division of Oral and Maxillofacial Surgery, Faculty of Dentistry, The University of Hong Kong, Hong Kong, China; lei0302@hku.hk (L.W.); richsu@hku.hk (Y.S.)

**Keywords:** melatonin, oral squamous cell carcinomas, invasion, migration, fibroblast growth factor 19 (FGF19), fibroblast growth factor receptor 4 (FGFR4)

## Abstract

Oral squamous cell carcinomas (OSCCs) are one of the most prevalent malignancies, with a low five-year survival rate, thus warranting more effective drugs or therapy to improve treatment outcomes. Melatonin has been demonstrated to exhibit oncostatic effects. In this study, we explored the anti-cancer effects of melatonin on OSCCs and the underlying mechanisms. A human tongue squamous cell carcinoma cell line (SCC-15) was treated with 2 mM melatonin, followed by transwell migration and invasion assays. Relative expression levels of Fibroblast Growth Factor 19 (FGF19) was identified by Cytokine Array and further verified by qPCR and Western blot. Overexpression and downregulation of FGF19 were obtained by adding exogenous hFGF19 and FGF19 shRNA lentivirus, respectively. Invasion and migration abilities of SCC-15 cells were suppressed by melatonin, in parallel with the decreased FGF19/FGFR4 expression level. Exogenous hFGF19 eliminated the inhibitory effects of melatonin on SCC-15 cells invasion and migration, while FGF19 knocking-down showed similar inhibitory activities with melatonin. This study proves that melatonin suppresses SCC-15 cells invasion and migration through blocking the FGF19/FGFR4 pathway, which enriches our knowledge on the anticancer effects of melatonin. Blocking the FGF19/FGFR4 pathway by melatonin could be a promising alternative for OSCCs prevention and management, which would facilitate further development of novel strategies to combat OSCCs.

## 1. Introduction

Cancer is one of the leading causes of death. There were over 18 million new cases and 9 million cancer-related deaths in 2018. Oral cancer was the 11th most common cancer [1]. It has high morbidity and mortality and affects nearly 600,000 people worldwide each year [2], among which 90% are oral squamous cell carcinomas (OSCCs) [3]. To date, surgery and adjuvant chemo-/radio-therapy have been the most widely used treatments for OSCCs. Despite the advances in technology for these treatment modalities in the past decade, the five-year survival rate of oral cancers remains low due to their rapid growth, local invasiveness, and distant metastasis [4]. Thus, exploring the underlying mechanisms of oral cancer metastasis and developing a potential therapeutic agent for oral cancer are imperative.

Melatonin, an endogenous hormone mainly produced by the pineal gland [5], has also been detected in multiple organs such as the brain, gut, skin and ovaries [6]. It was first chemically identified as *N*-acetyl-5-methoxytryptamine in the 1960s [7]. Since then, many studies have described the relevance of melatonin to human pathology and physiology. At present, it is well acknowledged that melatonin has numerous benefits such as antioxidant, anti-inflammatory and immunomodulatory effects [8,9,10,11]. Furthermore, a number of studies have outlined the anticancer effects of melatonin during the treatment of cervical cancer [12], osteosarcoma [13], leukemia [14], and glioblastoma [15]. Current knowledge of how melatonin exerts its anticancer effects include: antioxidant, anti-angiogenic, anti-mutagenic, reducing cell proliferation and modulating immune system [16,17,18,19,20]. In addition, melatonin has been reported to reduce the side effects of chemotherapy and radiotherapy, increase the therapeutic efficacy, and overcome drug resistance [21,22,23,24]. It has been proved that melatonin inhibits oral cell viability and angiogenesis, which is beneficial to oral cancer prevention and treatment [25]. These make melatonin plausible to be used as an adjuvant therapy to prevent and treat cancer. Studies also showed that there are some signaling pathways related to melatonin activity in restraining cancer such as BAX/BAK (proapoptotic proteins), NF-κB (nuclear factor kappa B), JNK (c-Jun *N*-terminal kinase) and VEGF (vascular endothelial growth factor), which are still not fully understood [26]. Therefore, the possible molecular and signaling pathways involved need further investigations.

FGF19 (fibroblast growth factor 19) interacts with receptor tyrosine kinases (FGFR4) to exert its regulating effect on cell growth, differentiation, metabolism and morphogenesis [27]. The FGF19/FGFR4 signaling pathway dysregulation is widely detected in many types of diseases including cancers, especially in hepatocellular carcinoma (HCC). Studies showed that FGF19 is highly expressed in HCC, and it promotes proliferation and invasion abilities of HCC cells [28]. In addition, it has been demonstrated that FGF19/FGFR4 is critical for the development and progression of head and neck squamous cell carcinoma [29]. Since FGF19/FGFR4 signaling acts as an oncogenic pathway and is involved in the cancer progression, targeting FGF19/FGFR4 signaling might be an effective anticancer therapy.

In this study, the antimetastatic effect of melatonin in human tongue squamous carcinoma cells were investigated. To our knowledge, only very few studies investigated the effect of melatonin on oral cancer metastasis and migration. Thus, this study would enrich our understanding on the underlying mechanisms of oral cancer progression and provide more evidence for melatonin to be used as an adjuvant oral cancer treatment.

## 2. Results

### 2.1. Effects of Melatonin on Viability, Migration, and Invasion of SCC-15 Cells

**Figure 1:**
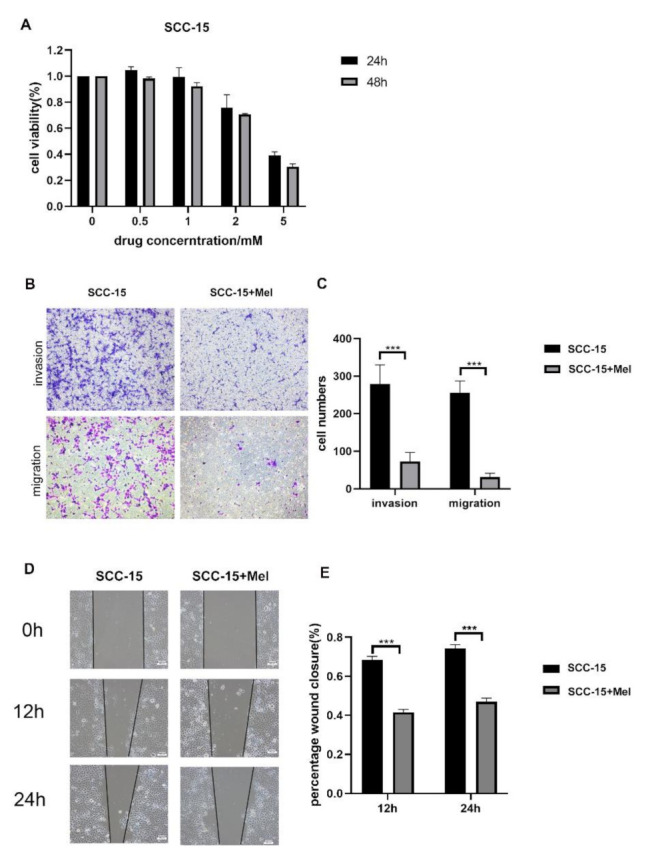
Effects of melatonin (Mel) on SCC-15 cells viability, invasion and migration. SCC-15 cells were treated with different concertation of Mel (0, 0.5, 1, 2, 5 mM) for 24 h and 48 h. (**A**): Cell viability tested by CCK-8. (**B**,**D**): SCC-15 cells were treated with or without 2 mM melatonin, the invasion and migration ability of cell was determined by trasnwell migration assays at 24 h (**B**), transwell invasion assays at 36 h (**B**) and wound healing at 12 h, 24 h (**D**). (**C**,**E**): Quantitative data from three independent experiments are shown in the right panels, respectively (*** *p* < 0.001). Scale bars: (**B**,**D**), 100 nm.

To assess the viability of SCC-15 cells under the stimulation of melatonin, CCK-8 assays were performed. The inhibition effect of melatonin on SCC-15 cells survival exhibited dose- and time-dependent manners, and the half maximal inhibitory concentration (IC50) was approximately 2 mM (Figure 1A). Therefore, 2 mM concentration of melatonin was chosen for further experiments. Wound healing assays were performed to test the effect of melatonin on SCC-15 cells migration (Figure 1D). Results showed that 2 mM melatonin sharply decreased the migration ability of SCC-15 cells at both 12 h and 24 h. In addition, transwell migration and invasion assays were performed. It was found that compared to the control group, SCC-15 cells treated with melatonin had significantly less cells invading the membrane, indicating that 2 mM melatonin significantly inhibited the migration and invasion of SCC-15 cells (Figure 1B).

### 2.2. Effect of Melatonin on FGF19/FGFR4 mRNA and Protein Expression

**Figure 2:**
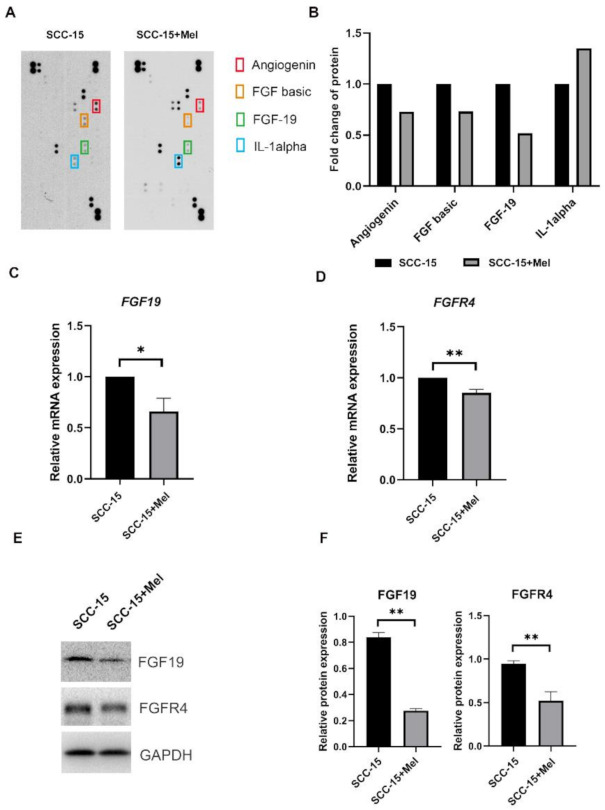
Melatonin-induced inhibition of FGF19 and FGFR4 expression (mRNAs and proteins) in Scheme 15. cells. Relative expression levels of 105 cytokines after being treated with 2 mM Mel or not for 24 h (**A**). Four cytokines with remarkable changes in intensity were highlighted (**B**). Expression levels of FGF19 and FGFR4 were determined by qPCR (**C**,**D**) and Western blot (**E**). The Western blot data were obtained from three independent replicates (**F**). * *p* < 0.05, ** *p* < 0.01.

To further elucidate the underlying mechanisms of melatonin on SCC-15 cells migration and invasion, cytokine arrays were used to test the inflammation cytokines expression level of SCC-15 cells with or without 2 mM melatonin. SCC-15 cells stimulated with 2 mM melatonin had a higher IL-1α and lower angiogenin, FGF basic and FGF19 protein expression level, compared to the control group (Figure 2A,B). Consistently, FGF19 mRNA and protein expression levels were further verified by RT-qPCR and Western blot, which showed that melatonin downregulated the expression of FGF19 and FGFR4 in SCC-15 cells (Figure 2C–E).

### 2.3. FGF19/FGFR4 Overexpression Increased the Invasion and Migration Abilities of SCC-15 Cells Attenuated by Melatonin

**Figure 3:**
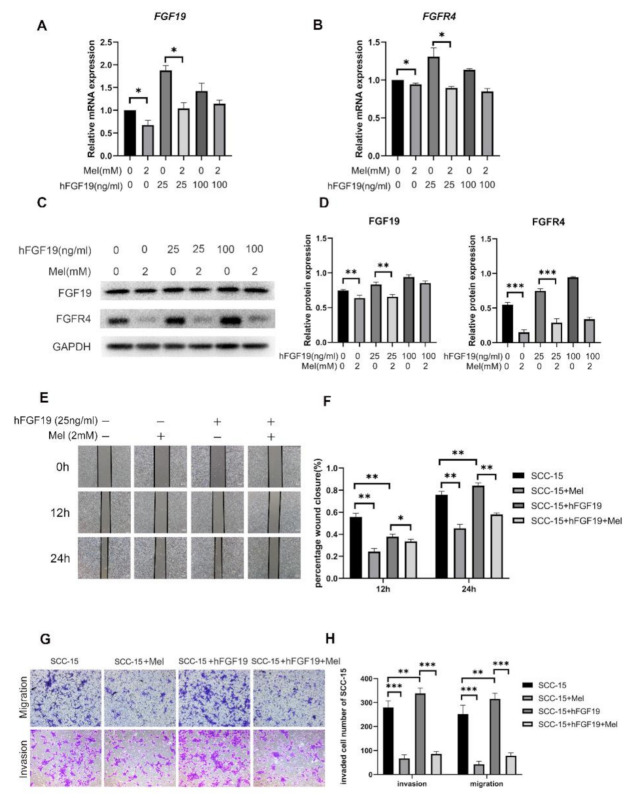
The effect of melatonin on cell migration and invasion in SCC-15 cells with or without FGF19 overexpression. The mRNA (**A**,**B**) and protein expression levels of FGF19/FGFR4 (**C**) after hFGF19 overexpression. The invasion and migration ability of cell was determined by trasnwell migration assays at 24 h (**G**), transwell invasion assays at 36 h (**G**) and wound healing at 12 h, 24 h, 36 h (**E**) (Scale bars: (**E**,**G**), 100 nm). Quantitative data from three independent experiments are shown in the right panels, respectively (**D**,**F**,**H**). * *p* < 0.05; ** *p* < 0.01, *** *p* < 0.001.

To further explore the function of FGF19/FGFR4 on SCC-15 cells during migration and invasion, FGF19 overexpression was conducted through adding exogenous hFGF19. We firstly demonstrated that hFGF19 did not affect the SCC-15 cells viability at 200 ng/mL for 48 h (Figure S1). Then, SCC-15 cells were treated with 25 ng/mL and 100 ng/mL hFGF19 for 24 h, with or without 2 mM melatonin afterwards. Results showed that after being treated with 25 ng/mL hFGF19 for 24 h, the mRNA and protein expression levels of FGF19 and FGFR4 were significantly increased, compared to the control group and the group treated with 100 ng/mL hFGF19. Besides, the group treated with both hFGF19 and melatonin had a lower expression level of FGF19 and FGFR4, compared to the group treated with hFGF19 alone (Figure 3A–C). In addition, wound healing, transwell invasion and migration assays confirmed that adding 25 ng/mL of hFGF19 increased the invasion and migration abilities of SCC-15 cells compared to the control group. Furthermore, the group treated with both hFGF19 and melatonin had markedly less cells invading the membranes than the group stimulated with hFGF19 only (Figure 3E,G).

### 2.4. Knocking down FGF19 Partially Strengthened the Suppression Effects of Melatonin on SCC-15 Cells Invasion and Migration

**Figure 4:**
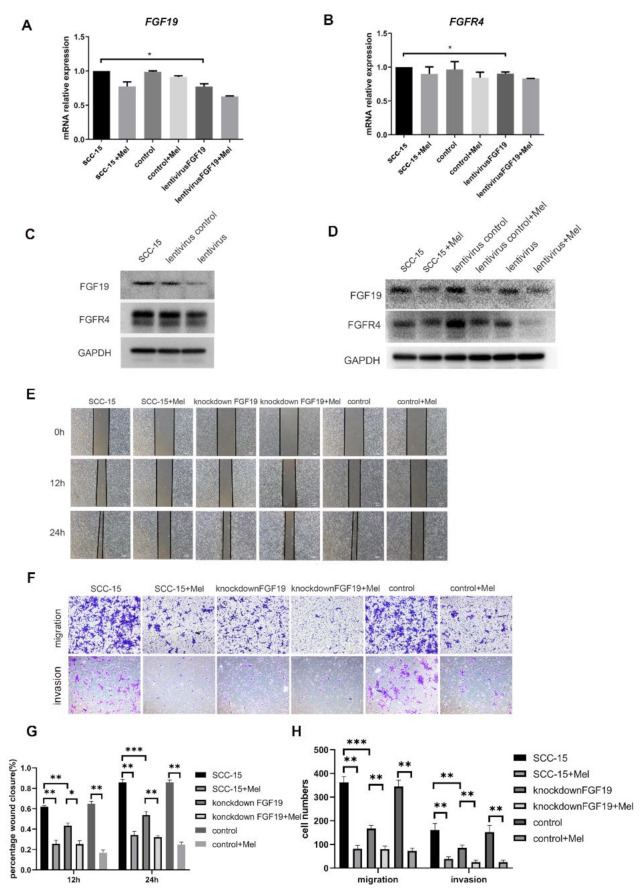
The mRNA and protein expression levels of FGF19/FGFR4 after FGF19 knocking down. The mRNA (**A**,**B**) and protein expression levels of FGF19/FGFR4 (**C**,**D**) after hFGF19 knocking down. The invasion and migration ability of cell was determined by trasnwell migration assays at 24 h (**F**), transwell invasion assays at 36 h (**F**) and wound healing at 12 h, 24 h, 36 h (**E**) (Scale bars: (**E**,**G**), 100 nm). Quantitative data from three independent experiments are shown in the bottom, respectively (**G**,**H**). * *p* < 0.05; ** *p* < 0.01, *** *p* < 0.001.

To verify the biological function of the FGF19/FGFR4 axis on oral cancer, SCC-15 cells were transfected with a sh-NC-Vector and a sh-FGF19-vector, which had no effects on the cell viability (Figure S2). Knocking down of FGF19 led to reduced FGF19 and FGFR4 mRNA and protein expression levels (Figure 4A–D). Furthermore, the migration and invasion abilities of SCC-15 cells treated with sh-FGF19-vector were decreased and the combined treatment of melatonin further suppressed cell migration and invasion (Figure 4E,F).

## 3. Discussion

The metastasis of cancer is generally observed at the terminal stage, which is the major cause of cancer-related death. Therefore, the agents that have the potential to prevent the migrative and invasive nature of cancer can open up novel therapeutic strategies. Epidemiological studies reported an increased cancer risk in individuals whose melatonin levels were compromised [30,31], thus leading to the investigations on the oncostatic effects of melatonin. An increasing number of studies showed that melatonin had an inhibiting effect on invasion and metastasis of different types of cancers, such as breast cancer [32], ovarian cancer, prostate cancer [33] and gastric cancer [34]. However, very little was found in the literature about the effect of melatonin on OSCCs migration and invasion. In this study, we demonstrated that melatonin suppressed oral cancer cells migration and invasion, which implied that melatonin might be a potential adjuvant therapeutic for oral cancer.

It has been demonstrated that the inhibitory effects of melatonin on cancer metastasis and invasion is by regulating several intracellular pathways including mitogen-activated protein kinase (MAPK), extracellular signal-regulated kinase (ERK) and protein kinase B (AKT/PKB) signaling pathways [35]. Nevertheless, very few studies explain this effect of melatonin on OSCCs and explore the underlying mechanisms. In the present study, we further identified the mechanisms of this inhibitory effect by conducting cytokine arrays to test the inflammation cytokines expression level of SCC-15 cells after being treated with melatonin. Our results showed that FGF19/FGFR4 were involved in the melatonin modulated oral cancer migration and invasion. Further verification showed that melatonin downregulated the mRNA and protein expression levels of FGF19 and FGFR4 in SCC-15 cells. Therefore, the mechanism for melatonin suppressing SCC-15 cells migration and invasion might be related to the FGF19/FGFR4 axis. According to previous studies, the FGF19/FGFR4 axis played a pivotal role in many different cancers such as hepatocellular carcinoma [36], breast cancer [37], ovarian cancer [38], and prostate cancer [39], which led to their low 5-year survival rates. These studies further demonstrated that FGF19/FGFR4 contributed to the invasion, metastasis, proliferation, and anti-apoptosis of cancer cells. Thus, targeting FGF19/FGFR4 could be a promising approach to combat cancers.

FGFRs inhibitor, and the monoclonal antibodies against FGF19/FGFR4 have been used to block the FGF19/FGFR4 signaling pathway. For example, fisogatinib (BLU-554) is a highly potent and selective FGFR4 inhibitor that has been used in clinical trials [40]. Desnoyers et al. [41] produced a neutralizing anti-FGF19 monoclonal antibody 1A6 and tested its function in FGF19 transgenic (FGF-TG) mice. U3-1784, a novel FGFR4 targeting antibody, exerts strong antitumor effects specific to liver cancer cells overexpressing FGF19 [42]. Notably, FGFR4 inhibitors also have direct effects on immune cells in the tumor microenvironment, such as myeloid-derived suppressor cells (MDSCs) and M2-type tumor associating macrophages (M2-TAMs). They suppress tumor progression through modulating paracrine signaling, immune evasion and angiogenesis [43]. Moreover, the simultaneous targeting of FGFR4 and other receptor tyrosine kinases (RTKs) has been shown to indirectly enhance anti-tumor activity through normalization of the tumor microenvironment [44]. This evidence make combination of FGFR4 inhibitors and immune therapy a feasible method for cancer patients.

However, application of FGFR4 inhibitor or anti-FGF19 antibody may cause unwanted adverse effects such as gastrointestinal side effects or dose-related liver toxicity [45]. Some tumors with FGFR4 mutation also showed resistance to the drugs [46]. Melatonin, an endogenous hormone, was demonstrated to suppress SCC-15 cells migration and invasion by blocking the FGF19/FGFR4 signaling pathway in this study. Besides the anticancer effects, melatonin has a favorable safety profile without major adverse events [47]. Not only does it reduce the toxic consequences of anti-cancer drugs, but it also increases their efficacy by enhancing the sensitivity of cancer cells to conventional anticancer drugs, even rendering resistant cancer cells to sensitive ones [19]. These suggest that melatonin may have synergistic anti-cancer functions with FGF19 and FGFR4 inhibitors.

FGF19/FGFR4 have been found to interact with several intracellular signaling pathways, including signal transducer and activator of transcription 3 (STAT3), mammalian target of rapamycin (mTOR), Jun *N-*terminal kinase (JNK), phosphoinositide 3-kinase (PI3K), β-catenin, and extracellular regulated protein kinase (ERK) [48]. Thus, it is possible that other signaling pathways might also be involved in the inhibitory effects of melatonin on FGF19/FGFR4. Further investigations are needed to explore more details about the melatonin-FGF19/FGFR4 network, which may help us to obtain a better understanding on cancer progression and find more approaches to tackle malignant tumors, not only in the head and neck but also in other systems.

## 4. Materials and Methods

### 4.1. CELL Line and Cell Culture

Human tongue squamous cell carcinoma cell line SCC-15 (ATCC Cat# CRL-1623, RRID: CVCL1681ATCC), a widely used cell lines for the in vitro study of oral cancer, was chosen for this study. SCC-15 cells were cultured in DMEM/F12 medium (Dulbecco’s Modified Eagle Medium: Nutrient Mixture F-12 Gibco, Thermo Scientific, Waltham, MA, USA), supplied with 10% Fetal Bovine Serum (FBS), 100 IU/mL penicillin, 100 μg/mL streptomycin and 400 ng/mL hydrocortisone (Sigma-Aldrich, St. Louis, MO, USA) at 37 °C in a humidified atmosphere with 5% CO_2_. Then, 2 M Melatonin solution (St. Louis, MO, USA) was first dissolved in DMSO (suspending liquid) and then diluted in the medium to a working concentration (2 mM).

### 4.2. Cell Viability Assay

Cell viability was evaluated using a CCK-8 assay (Dojindo, Tokyo, Japan) according to the product instruction. In brief, cells were seeded into 96-well plates (5000 each well) and treated with different concentrations of melatonin (0, 0.5, 1, 2, 5 mM). After incubating at 37 °C in a humidified atmosphere of 5% CO_2_ for 24 h and 48 h, 10 μL of CCK-8 reagent was added into the wells. The plate absorbance at 450 nm was detected by a SpectraMax M2 microplate reader (Molecular Device, San Jose, CA, USA) after incubating the plates for 4 h at 37 °C.

### 4.3. Cytokine Array—Human Cytokine Antibody Array

Cell lysates were collected, and the total protein concentration of each lysate was determined using the BCA method. Lysates from three independent repeats were pooled for the assay. Cytokine assays were conducted with a human XL cytokine array kit (R&D, Minneapolis, MN, USA) according to the standard procedures; the chemiluminescence were detected by ChemiDoc™ Touch Imaging System with Image Lab™ Touch Software 6.1 (Bio-Rad, Hercules, CA, USA). Images were analyzed by HLImage++ software 6.2 (Western Vision Software, Salt Lake City, UT, USA).

### 4.4. Quantitative Real Time- Polymerase Chain Reaction (qRT-PCR)

Total RNA was extracted from the SCC-15 cells using a TaKaRa MiniBEST Universal RNA Extraction Kit (TaKaRa, Shiga, Japan) according to the manufacturer’s instructions. Reverse transcription of extracted RNA into Complementary DNA (cDNA) was carried out using PrimeScript RT reagent Kit (TaKaRa, Shiga, Japan). qRT-PCR was performed using the TB Green Premix DimerEraser (TaKaRa, Shiga, Japan) in an ABI 7500 real-time PCR instrument. Gene expression of SCC-15 cells was normalized to β-actin. All the relative expression levels were calculated according to the 2−ΔΔCt method. The primers used for qRT-PCR are as follows: FGF19: forward 5′-CCGACGGCAAGATGCA-3′, reverse 5′-TCCTCCTCGAAAGCACAGTCT-3′; FGFR4: forward 5′-CCATAGGGACCCCTCGAATAG-3′, reverse 5′- CAGCGGAACTTGACGGTGT-3′; β-actin: forward 5′-TTGGCAATGAGCGGTT -3′, reverse 5′- AGTTGAAGGTAGTTTCGTGGAT -3′.

### 4.5. Trans well Migration and Invasion Assays

SCC-15 cells with different treatments were collected in 200 μL serum-free medium, and 5 × 10^4^ SCC-15 cells of each group were seeded in the upper chamber using 8 μm, 24-well insert Transwell chambers (Corning Inc., Lowell, MA, USA) with a polycarbonate membrane (pre-coated with Matrigel in invasion assay). After adding 750 μL medium with 10% serum in the lower chamber, cells were incubated for 24 h (migration) and 36 h (invasion) separately. After removing the cells on the top of the membrane using a cotton swab, the remaining cells were fixed with 4% paraformaldehyde, permeabilized with 100% methanol, stained with crystal violet, and photos were taken with a microscope.

### 4.6. Wound Healing Assay

SCC-15 cells with different treatments were collected and seeded into six-well plates at 95% confluence. After being cultured with serum starvation (0% FBS) for 24 h, a scratch wound was made using the 200 µL pipette tube at the bottom of the well. Then, the cells were cultured with the medium supplemented with 2% FBS. Images about the cell migration were captured at 0, 12 h, 24 h at the marked region with the microscope.

### 4.7. Western Blot Analysis

Cells of different groups were collected for total protein extraction. An equal quantity of proteins was separated by 10% sodium dodecyl sulfate-polyacrylamide gel electrophoresis (SDS-PAGE) and transferred onto polyvinylidene fluoride (PVDF) membranes. After being blocked with 5% skimmed milk in PBST for 2 h, the membranes were incubated with primary antibodies (1:1000) at 4 °C overnight. Then, the membranes were washed and incubated with anti-rabbit horseradish peroxidase (HRP)-linked secondary antibodies (1:5000) for 2 h at room temperature, and the blots were visualized using ECL hypersensitive luminescence (Thermo Scientific, Waltham, MA., USA). The primary antibodies FGF19 (D1N3R) (Rabbit mAb #83348), FGF Receptor 4 (D3B12) XP^®^ (Rabbit mAb #8562) were bought from Cell Signaling Technology (Danvers, MA, USA).

### 4.8. shRNA Lentiviral Particles Transduction

Prior to transfection, SCC-15 cells were seeded into 6-well plates (2 × 10^5^ cells/well) and cultured for 1 day until the cells reached 60–80% confluence. Then, the cells were transfected with control shRNA or FGF-19 shRNA lentiviral particles. All transfections were performed following the protocol of shRNA lentiviral particle transduction. Stable clones expressing the shRNA were selected via puromycin dihydrochloride, and the selected cells were expanded and collected to conduct shRNA expression analysis by Western Blot and qRT-PCR. Puromycin dihydrochloride (sc-108071), control shRNA (sc-108080), and FGF-19 shRNA lentiviral particles (sc-39480-V) were purchased from Santa Cruz Biotechnology, Inc. (Heidelberg, Germany).

### 4.9. Statistical Analysis

Each experiment was repeated independently at least three times and data were analyzed using the GraphPad Prism 9.2.0. (GraphPad Software, San Diego, CA, USA) and are represented as mean ± SD. The student’s t-test was used for the comparisons between two groups, and one-way ANOVA was used for the comparisons of three or more groups with one independent variable. *p*-values < 0.05 were considered to be statistically significant (* *p* < 0.05; ** *p* < 0.01; *** *p* < 0.001).

## 5. Conclusions

In conclusion, the present study demonstrates that melatonin suppresses oral squamous cell carcinomas invasion and migration through blocking FGF19/FGFR4 pathway. Melatonin, as a widely used health product, exhibits powerful anticancer effects in vitro. The FGF19/FGFR4 pathway could be an alternative target for cancer prevention and management. Further investigations are needed to explore more applications of melatonin in vivo. Our finding may break new ground on cancer management, which could inspire us to develop novel approaches to managing other malignant diseases using endogenous hormones.

## Data Availability

Not applicable.

## Supplementary Materials

The following are available online at https://www.mdpi.com/article/10.3390/ijms22189907/s1.
