# Supplementary material for: Melatonin Suppresses Oral Squamous Cell Carcinomas Migration and Invasion through Blocking FGF19/FGFR 4 Signaling Pathway
Source: Int J Mol Sci. 2021 Sep 14;22(18):9907. doi: 10.3390/ijms22189907 (PMC8468793; doi:10.3390/ijms22189907)
Supplement: Supplementary file 1 [file ijms-22-09907-s001.zip › ijms-1322188-supplementary.pdf]

Supplementary Materials:

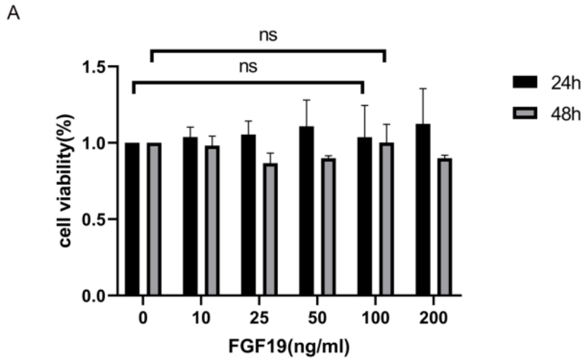

Figure S1. Effects of hFGF19 on SCC-15 cells. SCC-15 cells were treated with different concertation of hFGF19 (0, 10, 25, 50, 100, 200 ng/ml) for 24 h and 48 h. (A): Cell viability tested by CCK-8.

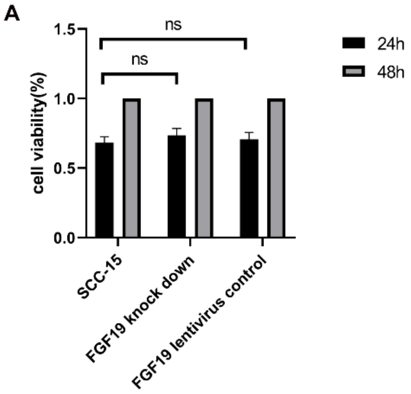

Figure S2. Cell viability of SCC-15 cells transfected with sh-NC-Vector and sh-FGF19-vector. SCC-15 cells, SCC-15 cells transfected with sh-NC-Vector and sh-FGF19-vector were incubated for 24 h and 48 h. (A): Cell viability tested by CCK-8.
